# Impact of COVID-19 Self-Isolation on Medical Students’ Education and Adherence to Protective Measures

**DOI:** 10.4269/ajtmh.21-1046

**Published:** 2022-04-11

**Authors:** Fida Thekrallah, Saif Aldeen AlRyalat, Ahmad Qarajeh, Akram Kilani, Dana AlQatawneh, Eman Badran, Ayman Qatawneh

**Affiliations:** ^1^ Department of Obstetrics and Gynecology, School of Medicine, The University of Jordan, Amman, Jordan;; ^2^ Department of Ophthalmology, School of Medicine, The University of Jordan, Amman, Jordan;; ^3^ School of Medicine, The University of Jordan, Amman, Jordan;; ^4^ Department of Pediatrics, School of Medicine, The University of Jordan, Amman, Jordan

## Abstract

This study aims to evaluate the impact of self-isolation on the level of adherence to health protective measures among medical students in Jordan and on their clinical education. Because of being suspected of having or testing positive for COVID-19, 336 students were self-isolated . A questionnaire was sent to study the clinical adherence of students to COVID-19 protective measures after their self-isolation period, the student’s satisfaction about the policy followed during the pandemic, the impact of these measures on their clinical training, and the level of vaccine acceptance among them. The study included 283 participants, with a mean age of 22.5 (±1.50) years; 49.5% males and 50.5% females. We found that students’ adherence to protective measures generally increased after their self-isolation. Gender, age, and having an infection from the hospital were the most important predictors for better adherence to health safety measures. Most students (83%) have registered to take the vaccine. 97.5% of self-isolated students reported that they are aware and satisfied of the School of Medicine instructions and policies. The findings suggest the need to ensure that medical students’ clinical training should not be negatively affected by COVID-19 and COVID-19 self-isolation, as medical students are adherent to COVID-19 precautionary measures and willing to take the vaccine.

## INTRODUCTION

The first cases of COVID-19 were identified in Wuhan, China, in December 2019^1^ and the WHO has officially declared COVID-19 as a pandemic on March 11, 2020.^2^ As of May 18, 2021, there have been around 163 million confirmed cases of COVID-19 globally and more than 725,000 confirmed cases of COVID-19 in Jordan.^3^

This highly contagious virus spreads through direct and indirect contact that led to the rapid spread of the disease and pushed countries worldwide including Jordan to implement interventions such as social distancing, the obligatory use of face masks, the use of disinfectants, national curfews, and quarantines.^4^

This pandemic has significantly affected medical students’ clinical training as some institutions suspended their clinical rotations because of safety concerns.^5^ Moreover, the self-isolation of medical students led to missing more clinical rotations. Noting that the medical students’ self-isolation protocol was the same as any COVID-19 case, as identified in the national protocols by the Ministry of Health in Jordan.

Medical students’ adherence to precautionary measures along with proper infection control training are effective methods to help decrease self-isolation days and the number of lost clinical rotations. Few studies examined medical students’ adherence to face masks as compared with nonmedical students,^6^ and another study looked at the precautionary measures adopted by medical students in Jordan.^7^ However, data on the change in adherence to COVID-19 precautions after self-isolation as compared with before self-isolation among medical students is scarce. Therefore, we sought to assess the impact of self-isolation on the level of adherence to health protective measures among medical students in Jordan. We also examined the impact of self-isolation on their clinical education. It is important for us to fill this gap and understand the impact self-isolation has on education and the adherence to protective measures to help people in charge implement new instructions to minimize the effect the pandemic is causing as it is still ongoing.

## MATERIALS AND METHODS

### Participants.

Since the beginning of the 2020–2021 academic year on September 27, 2020, the School of Medicine at the University of Jordan has put in place a clear plan to deal with the COVID-19 pandemic. The Ministry of Health of Jordan suspended all in-class lectures and activities for all except the clinical year students of medicine and dentistry. They gave these faculties the option to resume clinical training during the pandemic with special restrictions that were reviewed by the Ministry of Health or suspend all training. The impact of the suspension of all training will do on medical education and the future of the healthcare system (e.g., shortage of clinicians, increased unnecessary consultations and decreased quality of care, skills, and knowledge) would have been massive. Weighing the benefits and risks, the Faculty of Medicine of the University of Jordan decided to suspend all in-class lectures and seminars and resumed them as online lectures; however, clinical rotations were not canceled but, the hours that students spent at the hospital has been reduced. All students were required to wear face masks, adhere to public safety measures, and head home immediately after their clinical rotations.

Despite that, the infection of medical students was inevitable. Therefore, instructions were set regarding the self-isolation of students who got infected, developed symptoms of COVID-19 infection, got in close contact with a COVID-19 case or with a suspected COVID-19 case, or due to travel regulations. These self-isolation protocols followed were the same as any COVID-19 case as identified in the national protocols by the Ministry of Health in Jordan. The School of Medicine instructed any student with a positive COVID-19 test result to self-isolate themselves for 14 days, and anyone who got in close contact with a COVID-19 patient (family member, friends, roommate, and hospital inpatient) to self-isolate for 10 days and then return to their clinical rotations on the 11th day after obtaining a negative COVID-19 PCR test result. If a student who came in contact with a COVID-19 patient developed symptoms before their 10th day, they have to undergo a COVID-19 test when symptoms appear. When a student comes across a COVID-19 case but are not in close contact, they continue their rotations while adhering to public safety measures. For the cases in which it is difficult to determine the status of contagiousness, or if there are suspicious symptoms of infection despite a negative COVID-19 test, the Infection Control Office at the Jordan University Hospital and the respiratory disease specialist are consulted to ensure that the right decisions are being made.

All clinical students were informed about these instructions, and the students were given a contact number with which they could directly communicate at any time; the contact number belonged to the Assistant Dean for Clinical Training and Graduate Affairs, who is also a physician. The contact number accepted calls and messages 24/7; students called to update on their health and infection status, their symptoms, their COVID-19 test results.

The communication continued with the students to check on their condition regularly, to inform them on their period of self-isolation, to update them on their compensation days for their missed days of clinical training due to isolation, and the rescheduling of their exams if they overlapped with their self-isolation period. The clinical departments were informed continuously about the names of the self-isolated students, the period of their self-isolation, and the plan for compensation of missed clinical training and their examinations. The information about any self-isolated student was added instantaneously to a data sheet. The data sheet consisted of the students who were self-isolated due to COVID-19 infection, developing symptoms of COVID-19, getting in contact with a COVID-19 case, or due to travel regulations during the time period of September 27, 2020 to March 9, 2021. On March 9, the Ministry of Higher Education decided to suspend all clinical training and proceed with online teaching due to the high number of COVID-19 cases in Jordan.

The authors constructed a questionnaire in English to study the impact of their self-isolation period on their clinical adherence to health protective measures, the student’s satisfaction with the policy that was followed during the pandemic, the impact of these measures on their clinical training, and the level of vaccine acceptance among them. The IRB approval was received from the Scientific Research Committee at the School of Medicine/UJ and the Jordan University Hospital.

The questionnaire was made on Google Forms and consisted of four parts: participants’ consent, demographics, self-isolation and adherence to protective measures, and clinical education.

The total number of medical students in clinical years in the 2020–2021 academic year is 1,207 students: 428 in their fourth year, 403 in their fifth year, 376 in their sixth year. The participants’ inclusion criteria were the medical students in their clinical years at the University of Jordan who got self-isolated in the period from September 27, 2020 to March 9, 2021, due to infection with a positive COVID-19 result, developing symptoms of COVID-19, getting in close contact with a known or suspected COVID-19 patient, or due to travel regulations. The number of students who have fulfilled the inclusion criteria was 336. The Google Form questionnaire was sent to all the 336 students through WhatsApp on April 20, 2021. The form was closed on April 24, 2021. Consent was obtained from all participants. The response rate was 84%; as 283 out of the 336 students completed all the sections of the survey and were included in the study.

### Variables.

The questionnaire consisted of four sections. In the first section, we obtained consent. The second section included questions about demographics; age, gender, year of study, and who they lived with. It also inquired about the number of times and the cause of self-isolation or infection in addition to the source of the students’ COVID-19 infection and close contact whether it was from the hospital or other places.

While in the third section, we have investigated the acceptance rate of the COVID-19 vaccine and the main purpose for registering to take it among the students. Moreover, in this section, we have calculated the adherence score by combining the scores for the following five questions that asked about measures taken to protect against COVID-19 infection:

“After self-isolation, how was your adherence to the following affected, compared to the time before your self-isolation”:

1. Wearing a facemask properly
2. Practicing social distancing
3. Hand hygiene
4. Practicing protective measures with patients
5. Fear of getting infected/reinfected.

The maximum score was 25, representing a maximum increase in adherence to all measures, and the minimum score was 5, representing minimum adherence to protective measures.

The last section scaled the students’ satisfaction and awareness about the policy of the school of medicine regarding self-isolation using a Likert scale and checked if there was an efficient communication plan with the school. In the final part of the last section, we have investigated where the students did the COVID-19 test, the reason for the test whether it was a school of medicine request or personal request, or other causes, and how many days the students missed due to self-isolation.

### Statistical analysis.

We used SPSS v. 26.0 (Chicago, IL) in our analysis. We used mean (±SD) to describe continuous variables. We used count (frequency) to describe other nominal variables.

We have performed scale reliability analysis to find the Cronbach’s alpha of the scale. We have performed linear regression analysis to find predictors for adherence level, where we included only variables that were found to be significant on univariate analysis. We have reported the results mainly via the standardized beta coefficient.

All underlying assumptions were met. We adopted a *P* value of 0.05 as a significant threshold.

## RESULTS

A total of 283 participants were included in this study, with a mean age of 22.5 (±1.50) years. They were 140 (49.5%) men and 143 (50.5%) women. Characteristics of the included sample are detailed in Table 1.

**Table 1:** Details of the characteristics of included sample

		Mean	Standard deviation	Count	Column *N* %
Age		22.53	1.15	–	–
Gender	Male	–	–	140	49.5%
	Female	–	–	143	50.5%
Year of study	Fourth year	–	–	107	37.8%
	Fifth year	–	–	101	35.7%
	Sixth year	–	–	75	26.5%
Who do you live with?	Alone	–	–	31	11.0%
	With family	–	–	230	81.3%
	With colleagues	–	–	22	7.8%
How many times have you been self-isolated since the beginning of clinical training year 2020–2021?	Once	–	–	165	58.3%
	Twice	–	–	81	28.6%
	Three times	–	–	29	10.2%
	Four times	–	–	7	2.5%
	Five times	–	–	1	0.4%
How many times did you get infected?	Zero	–	–	74	26.1%
	One time	–	–	204	72.1%
	Two times	–	–	5	1.8%
Approximately, how many days of your active clinical training did you miss due to self-isolation?		9.88	6.97	–	–
Management required during your covid infection?	I didn’t get infected	–	–	74	26.1%
	Home-based treatment	–	–	200	70.7%
	Emergency department visit	–	–	9	3.2%
	Hospital admission	–	–	0	0.0%

The health protective measure questions were found to be highly reliable with Cronbach’s alpha of 0.873. Table 2 details the reliability score for each item in the health protective measure questions. The mean adherence score for the included sample was 17.38 (SD 4.27). On the linear regression model, the following variables were found to be significant on univariate analysis and included in the model, including age (0.009), gender (< 0.001), cause of isolation being infected (< 0.001), cause of isolation being in contact with known COVID-19 case (0.005), source of infection being from the hospital (0.037), and if management required for the COVID-19 infection (0.002). The model was significant (< 0.001), with an adjusted R square of 14.8%. Age (*P* = 0.015, beta = 0.14, B = 0.50 [0.10–0.91]), gender (*P* < 0.001, beta = 0.30, B = 2.53 [1.61–3.46]), and if the source being hospital (*P* = 0.02, beta = 0.13, B = 1.16 [0.18–2.14]) were the only significant predictors for adherence after isolation as shown in Table 3.

**Table 2:** The reliability score for each item in the health protective measure questions

	Scale mean if item deleted	Scale variance if item deleted	Corrected item-total correlation	Cronbach’s alpha if item deleted
After self-isolation, how was your adherence to wearing a facemask properly affected, compared with the time before your self-isolation:	13.88	11.659	0.805	0.821
After self-isolation, how was your adherence to practicing social distancing affected, compared with the time before your self-isolation:	14.01	11.695	0.795	0.824
After self-isolation, how was your adherence to hand hygiene affected, compared with the time before your self-isolation:	13.80	12.117	0.738	0.838
After self-isolation, how was your adherence to the practicing protective measures with patients affected, compared with the time before your self-isolation:	13.59	13.079	0.663	0.856
After self-isolation, how was your fear of getting infected/reinfected affected, compared with the time before your self-isolation:	14.24	11.681	0.565	0.892

**Table 3:** Predictors for adherence score

Model		Unstandardized coefficients		Standardized coefficients	t	Sig.	95.0% confidence interval for *B*	
		B	Std. error	Beta			Lower bound	Upper bound
1	(Constant)	2.959	4.916		0.602	0.548	−6.718	12.636
	Age	0.501	0.206	0.135	2.436	0.015	0.096	0.906
	Gender	2.534	0.469	0.297	5.401	0.000	1.610	3.458
	Cause: infected with a positive COVID-19 result	−0.991	0.859	−0.109	−1.153	0.250	−2.682	0.701
	Cause: got in contact with a known COVID-19 patient	0.823	0.572	0.095	1.438	0.151	−0.303	1.949
	Source: hospital (in university)	1.161	0.496	0.131	2.339	0.020	0.184	2.137
	Management required during your COVID infection?	−0.513	0.780	−0.059	−0.658	0.511	−2.049	1.022

*Dependent Variable: Adherence_score.

The majority of students included have registered to take the vaccine, where 235 (83%) registered compared with 48 (17%) who did not. For those who registered, 185 stated that they registered to prevent reinfection, whereas 50 registered for future travel and regulatory proposes.

Regarding the opinion of students on the school of medicine policy and their satisfaction, 97.5% (*N* = 276) of self-isolated students reported that they are aware of the School of Medicine instructions and policy regarding COVID-19 self-isolation of students and clinical training compensation. Upon their own personal request or personal suspicion of infection, 243 of the students (85.9%) performed the COVID-19 test; 12% (*N* = 34) performed the test upon the School of Medicine’s request, and six students (2.1%) has other reasons for performing the test. It was believed by 90.5% of the participants (*N* = 256) that there was an efficient plan of communication with the School of Medicine. One hundred and thirty-one participants (46.3%) reported that they have been compensated with all hours of clinical training they missed. Due to self-isolation, 53.8% reported that they have not been fully compensated for the missed clinical training days. Table 4 shows the responses of the self-isolated students regarding School of Medicine instructions and policy, clinical rotation and exam rescheduling, and their clinical education.

**Table 4:** Participants responses regarding their opinion on their clinical training

		Count	Column *N* %
How satisfied were you about school of medicine policy regarding self-isolated students?	Very unsatisfied	13	4.6%
	Unsatisfied	24	8.5%
	Neutral	78	27.6%
	Satisfied	125	44.2%
	Very satisfied	43	15.2%
How satisfied were you about clinical rotation rescheduling (compensation of the absent days)?	Very unsatisfied	33	11.7%
	Unsatisfied	58	20.5%
	Neutral	89	31.4%
	Satisfied	74	26.1%
	Very satisfied	29	10.2%
How satisfied were you about end of rotation exam rescheduling?	Very unsatisfied	33	11.7%
	Unsatisfied	32	11.3%
	Neutral	118	41.7%
	Satisfied	62	21.9%
	Very satisfied	38	13.4%
How satisfied were you about your clinical education and training?	Very unsatisfied	64	22.6%
	Unsatisfied	83	29.3%
	Neutral	78	27.6%
	Satisfied	47	16.6%
	Very satisfied	11	3.9%

## DISCUSSION

Our study showed that students’ adherence to protective measures generally increased after their self-isolation. On the regression analysis, we found that gender was the most important predictor for better adherence to health safety measures. The predictors of better adherence are being female (*P* < 0.001), increasing age (*P* = 0.009), and having an infection from the hospital (*P* = 0.037). A study on dental students in Saudi Arabia showed that females had more knowledge regarding COVID-19 that could be why, in our study, females had more adherence after their quarantine.^8^ Older medical students are more mature and have more clinical experience, thus they are more adherent to protective measures after self-isolation. Regarding getting infected from the hospital, the increased adherence after self-isolation might result from the fear of COVID-19 patients accommodation units and undiagnosed COVID-19 patients. It was found that medical students, in general, have a good level of knowledge about COVID-19 and implemented proper protective measures in Afghanistan, China, UAE, Pakistan, and Jordan.^7^^,^^9^^–^^12^ A study in Jordan focused on the medical students’ knowledge and attitude toward COVID-19 precautionary measures; they found that more than 80% of Jordanian medical students adopted social distancing and used hand disinfectants.^7^ Our study focused on medical students in the University of Jordan who got self-isolated. It showed that their adherence to protective measures, such as wearing face masks, hand wash, and social distancing, has increased after their period of self-isolation. One study conducted in Poland compared the facemask use between medical and nonmedical students and found that medical students were more adherent to face mask use.^6^

The majority of students (83%) included have registered to take the vaccine. For those who registered, 185 stated that they registered to prevent reinfection, whereas 50 registered for future travel and regulatory purposes. Two contraindicating studies conducted in India and Uganda discovered that vaccine acceptance among medical students was 89.4% and 37.3%, respectively.^13^^,^^14^ The self-isolated students at the University of Jordan have shown a high vaccine acceptance rate and most of them registered to prevent reinfection, thus preventing missing more clinical rotations as 53.8% of participants did not get fully compensated for their missed clinical training.

The students in their clinical years who have been self-isolated were shown to be aware of the School of Medicine instructions and policy and 90% of them believed that there was an efficient communication plan with the School of Medicine. Due to overlapping clinical rotation schedules, the limited number of students allowed per rotation, or because their self-isolation period was at the end of the semester, 53.8% of the students were not compensated for their missed clinical training. The students have shown high knowledge about COVID-19 infection as most of them performed the COVID-19 test upon their own personal suspicion of infection. A study in the United Kingdom showed that medical students preferred to continue clerkship training instead of online training despite the fear of the spread of the COVID-19 virus.^15^ The School of Medicine, University of Jordan’s instructions and policy, have been put to try to continue clinical training for the benefit of the students despite COVID-19 restrictions; the authors believe that the policy has been a successful one.

## LIMITATIONS

The limitations of our study were that our questionnaire was sent to the population via WhatsApp without interviewing them that could be a drawback due to over or underestimation of responses. Another limitation is that participants are subject to recall bias because the period of our study was wide about 7 months approximately. In addition, courtesy bias could be present in the responses as this is what students reported; their actual behavior was not studied.

## CONCLUSION

Our findings demonstrate increased adherence to protective measures after self-isolation and a high vaccine acceptance rate among Jordanian medical students. However, it was shown that almost half of the students did not get fully compensated for their missed clinical education. These results suggest the need to ensure that medical students’ clinical training should not be negatively affected by COVID-19 and COVID-19 self-isolation as most Jordanian medical students are adherent to COVID-19 precautionary measures and willing to take the vaccine.
